# Refractory Lumbar Pain Following Motor Vehicle Collision in a Geriatric Patient With Prior Lumbar Surgery: Clinical Resolution After Multimodal Conservative Spinal Structural Rehabilitation

**DOI:** 10.7759/cureus.103540

**Published:** 2026-02-13

**Authors:** Justin M Dick

**Affiliations:** 1 Chiropractic, Clear Life Scoliosis and Chiropractic Center, Charlotte, USA

**Keywords:** geriatric, motor vehicle collision, post-traumatic, spinal rehabilitation, surgical fusion

## Abstract

Motor vehicle collisions frequently result in persistent spinal pain in older adults, particularly among patients with a history of lumbar surgery, where altered biomechanics and compensatory loading can complicate recovery. In this population, care is often directed toward symptom management despite limited evidence for durable improvement and higher risks associated with invasive interventions. Reports of successful conservative structural rehabilitation remain relatively uncommon.

A 66-year-old female with previous lumbar fusion, cervical fusion, and mild left thoracic scoliosis presented with severe ongoing low back pain radiating into her lower extremity following a motor vehicle collision. This resulted in substantial functional limitation. After failing pharmacologic management and physical therapy, she underwent a 10-week multimodal conservative structural care program. This incorporated pulsed electromagnetic field therapy, extracorporeal shockwave therapy, and structural spinal rehabilitation. Post treatment, the patient demonstrated meaningful improvement, including reduced pain, improved function with Functional Rating Index scores decreasing from 22 to 11. The subject also reported resolution of radicular symptoms, independent unassisted ambulation, discontinuation of prescription analgesics, and radiographic improvement in sagittal alignment and spinal translation.

## Introduction

Motor vehicle collisions (MVCs) are a common cause of persistent spinal pain. Specifically, as adults grow older demonstrating higher rates of chronic symptoms and functional decline following trauma [1]. Age-related degenerative changes, reduced tissue elasticity, and diminished neuromuscular adaptability contribute to prolonged recovery trajectories in this population. When a history of prior lumbar surgery is present, post-collision lumbar pain often follows a more complex clinical course. This reflects altered biomechanics, preexisting structural compromise, and an increased susceptibility to persistent or refractory symptoms after trauma. This reflects altered spinal biomechanics and disrupted load distribution with compensatory movement strategies that increase susceptibility to refractory pain [2].

Lumbar pain following a motor vehicle collision would be assessed through a biomechanical and functional framework that accounts for prior surgical alteration. This approach also considers sagittal alignment and segmental stability when determining clinical significance and management. Early identification of mechanical pain drivers and targeted conservative intervention would be expected to reduce chronicity and limit escalation of care. Older patients with prior lumbar surgery are frequently managed through symptom-focused pathways. These emphasize pharmacologic treatment, generalized physical therapy, or interventional procedures, often without adequate structural assessment [3]. Persistent pain, in this population, is commonly attributed to irreversible postsurgical change rather than potentially modifiable mechanical dysfunction.

Existing literature indicates that patients with prior lumbar surgery experience higher rates of chronic low back pain and disability after secondary injury, including MVCs [4]. Post-surgical spines demonstrate altered kinematics and increased adjacent-segment loading. This may be exacerbated by acceleration-deceleration forces during trauma [5]. While revision surgery and interventional pain management are frequently considered in refractory cases, evidence supporting durable functional improvement in geriatric populations remains limited [6]. Conservative management is often recommended. This is inconsistently defined. It is rarely focused on restoring spinal alignment, postural control, or load tolerance in surgically altered spines.

The consequences of unresolved lumbar pain in older adults extend beyond pain severity alone. Persistent symptoms are associated with reduced mobility, increased fall risk, sleep disturbance, diminished quality of life, prolonged medication use, and increased healthcare utilization [7]. In patients with prior lumbar surgery, fear avoidance behaviors commonly develop following injury. Uncertainty regarding safe movement further contributes to functional limitation and prolonged disability [8]. There is a paucity of detailed case reports describing successful conservative structural rehabilitation in geriatric post-surgical patients following MVC.

This case report addresses this gap by describing a geriatric subject with a history of lumbar surgery who developed refractory lumbar pain following an MVC. These subjects experienced clinical resolution after a multimodal conservative structural care approach. This report aims to contribute clinically relevant evidence supporting conservative spinal structural care as a viable option in complex geriatric presentations.

## Case presentation

Institutional Review Board approval was not required for this report as it involved a retrospective review of de-identified data and met exemption criteria under the U.S. Common Rule (45 CFR 46.104). Written informed consent was obtained from the subject. No identifiable personal or protected health information is included in this report.

Subjects history

The subject of the case was a 66-year-old female presenting with persistent and debilitating low back pain following a motor vehicle collision on January 6, 2025. The subject's spinal health history included a previous lumbar laminectomy, lumbar spinal fusion (year 2022), cervical fusion (years 2006, 2009), and mild thoracic scoliosis. She had exhausted standard medical management pathways, including standard pharmacologic and physical therapy interventions, prior to entering the clinic. The subject stated she was working full-time but physically limited due to the pain, 8/10 severe level of pain on a standard 0-10 numeric pain rating scale.

The subjects' symptoms included low back pain (bilaterally), left hip pain, numbness and tingling in the left arm and leg, and persistent headache. The subject expressed that the back pain had been exacerbated since the motor vehicle accident. The subject denied loss of consciousness at the time of the incident and reports wearing a seatbelt. Pain levels were assessed using the Function Rating Index, which was administered at baseline and upon discharge. 

Physical examination and radiographs

The subject arrived at the office with a cane to assist in ambulation. Initial vital signs revealed a blood pressure of 156/89 mmHg with a heart rate of 69 beats per minute. The subject was advised to follow up with their primary care provider for further evaluation of elevated blood pressure. Handgrip dynamometry measured 31.2 pounds on the right and 26.4 pounds on the left. Peripheral oxygen saturation was 97%.

Postural examination revealed a forward head posture, right low shoulder, and anterior thoracic translation. Aberrant motion and intersegmental dysfunction with associated loss of normal movement were identified throughout the cervical, thoracic, and lumbar spine, as well as the pelvic region. Active range of motion was globally reduced in the cervical, thoracic, and lumbar spines, with pain reported throughout all planes of movement at each spinal region. Palpation demonstrated tenderness within the cervical, thoracic, and lumbar paraspinal musculature bilaterally, with areas of increased muscle tone and spasticity noted across the cervical, thoracic, lumbar, and pelvic regions. Tightness and tenderness were observed in the trapezius, cervical and thoracic paraspinals, rhomboids, quadratus lumborum, and lumbar paraspinal muscles bilaterally. The examination also revealed bilateral piriformis involvement, with greater pain reported on the left.

Cervical foraminal compression testing demonstrated bilateral neck pain without associated radicular symptoms. Spurling’s maneuver reproduced localized cervical pain, with the subject denying radiation into the upper extremities. Shoulder depressor testing similarly provoked bilateral neck pain without radiating symptoms. Kemp’s test reproduced lumbar pain accompanied by radiating symptoms, "whole left leg", as reported by the subject. Dermatomes and Myotomes were assessed bilaterally with a pinwheel (Table 1).

**Table 1:** Left-sided dermatomal and myotome findings on neurologic examination Neurologic examination revealed diffuse left-sided sensory abnormalities spanning the C5 through T1 dermatomes in the upper extremity. And, the L1 through S1 dermatomes in the lower extremity. Associated motor deficits were present on the left, with myotomal strength graded at 3/5 (N=5/5) across C5–C8 in the upper extremity. The lower extremity, L3–L5 strength in the remaining myotomes was preserved. Sensory and motor testing on the right side was unremarkable. The Functional Rating Index (FRI) combines components of the Oswestry Low Back Disability Questionnaire and the Neck Disability Index into a single outcome measure intended to reduce administrative burden. Scores range from 0 to 40, with higher scores indicating greater functional disability. The FRI has been previously validated and shown to demonstrate strong reliability, validity, and responsiveness in both clinical and research settings [9].

Spinal Level	06/03/2025 Dermatome Left	06/03/2025 Myotome Left	08/12/2025 Dermatome Left	8/12/2025 Myotome Left
C1	Intact	Intact	Intact	Intact
C2	Intact	Intact	Intact	Intact
C3	Intact	Intact	Intact	Intact
C4	Intact	Intact	Intact	Intact
C5	Positive	3/5	Intact	Intact
C6	Positive	3/5	Intact	Intact
C7	Positive	3/5	Intact	Intact
C8	Positive	3/5	Intact	Intact
T1	Positive	Intact	Intact	Intact
L1	Positive	Intact	Intact	Intact
L2	Positive	Intact	Intact	Intact
L3	Positive	3/5	Intact	Intact
L4	Positive	3/5	Intact	Intact
L5	Positive	3/5	Intact	Intact
S1	Positive	Intact	Intact	Intact
FRI	22		11	

**Figure 1:**
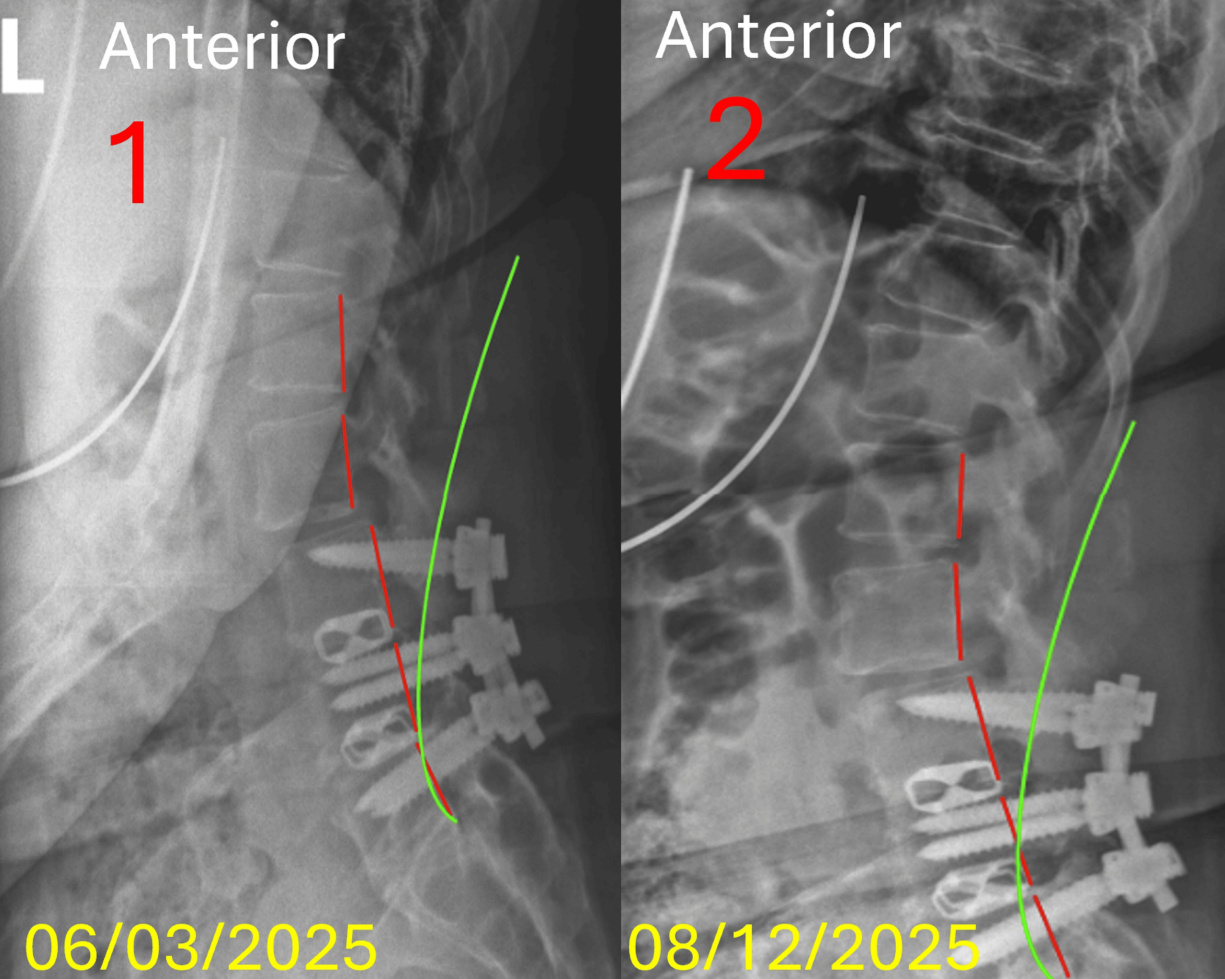
Lateral lumbopelvic radiograph comparison The green line depicts the expected alignment of the posterior longitudinal ligament in a normal spine. The red line represents the subjects' alignment as defined by George’s line, reflecting deviation of the posterior longitudinal ligament. (1) X-ray image represents the initial presentation to the office on 06/03/2025. Pre-treatment neutral lateral lumbar radiograph demonstrating a lumbar curvature (ARA L1-L5) of −27.1 and anterior translation (L1-S1) of 47.7 mm. (2) X-ray image represents the day of discharge from the office on 08/12/2025. Post-treatment neutral lateral lumbar radiograph showing improvement with ARA L1-L5 measuring −24.0 and L1-S1 measuring 30.0 mm. The Absolute Rotation Angle (ARA) tool is a radiographic measurement method used to assess the total angle of spinal curvature.

**Figure 2:**
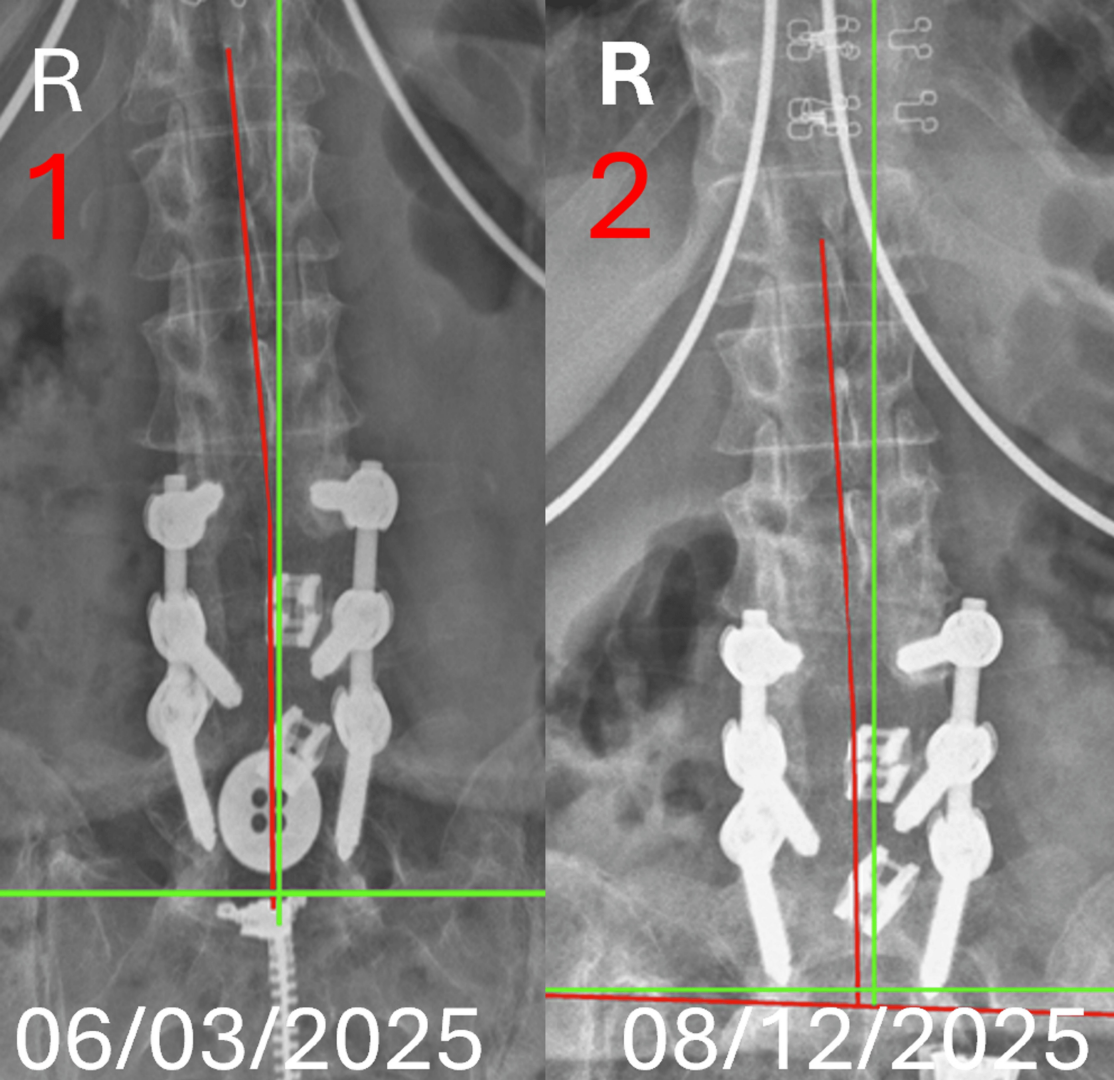
Anteroposterior (AP) lumbopelvic radiograph comparison The green line depicts the expected alignment of the posterior longitudinal ligament in a normal spine. The red line represents the subjects' alignment as defined by George’s line, reflecting deviation of the posterior longitudinal ligament. (1) X-ray image represents initial presentation to the office on 06/03/2025. Pre-treatment AP lumbar radiograph demonstrating translation of T12-S1 of -8.3 mm (normal is measured 0.0 mm). (2) X-ray image represents the day of discharge from the office on 08/12/2025. Post-treatment AP lumbar radiograph demonstrating translation of T12-S1 of -7.3 mm (normal is measured 0.0 mm).

Lumbar (anteroposterior (AP) and lateral) radiographs revealed a persistent right lateral thoracolumbar translation, decreased lumbar lordosis, and anterior thoracic translation. The images revealed evidence of post-surgical degenerative changes (Figures 1, 2). Radiographic imaging was repeated following the completion of the care plan. Follow-up radiographs were obtained with identical positioning and exposure settings to minimize variability and evaluated by the same physician as the initial films. Quantitative changes in lumbar lordosis and thoracolumbar translation were measured to assess structural outcomes utilizing PostureRay (PostureCo, Inc., Trinity, FL), a machine-learning-assisted parameter measurement. Prior studies have demonstrated high inter- and intra-examiner reliability of radiographic line-drawing methods with no significant differences compared to manual measurements across spinal regions. These metrics were compared with established normative data as well as baseline imaging to determine the degree of anatomical correction.

**Figure 3:**
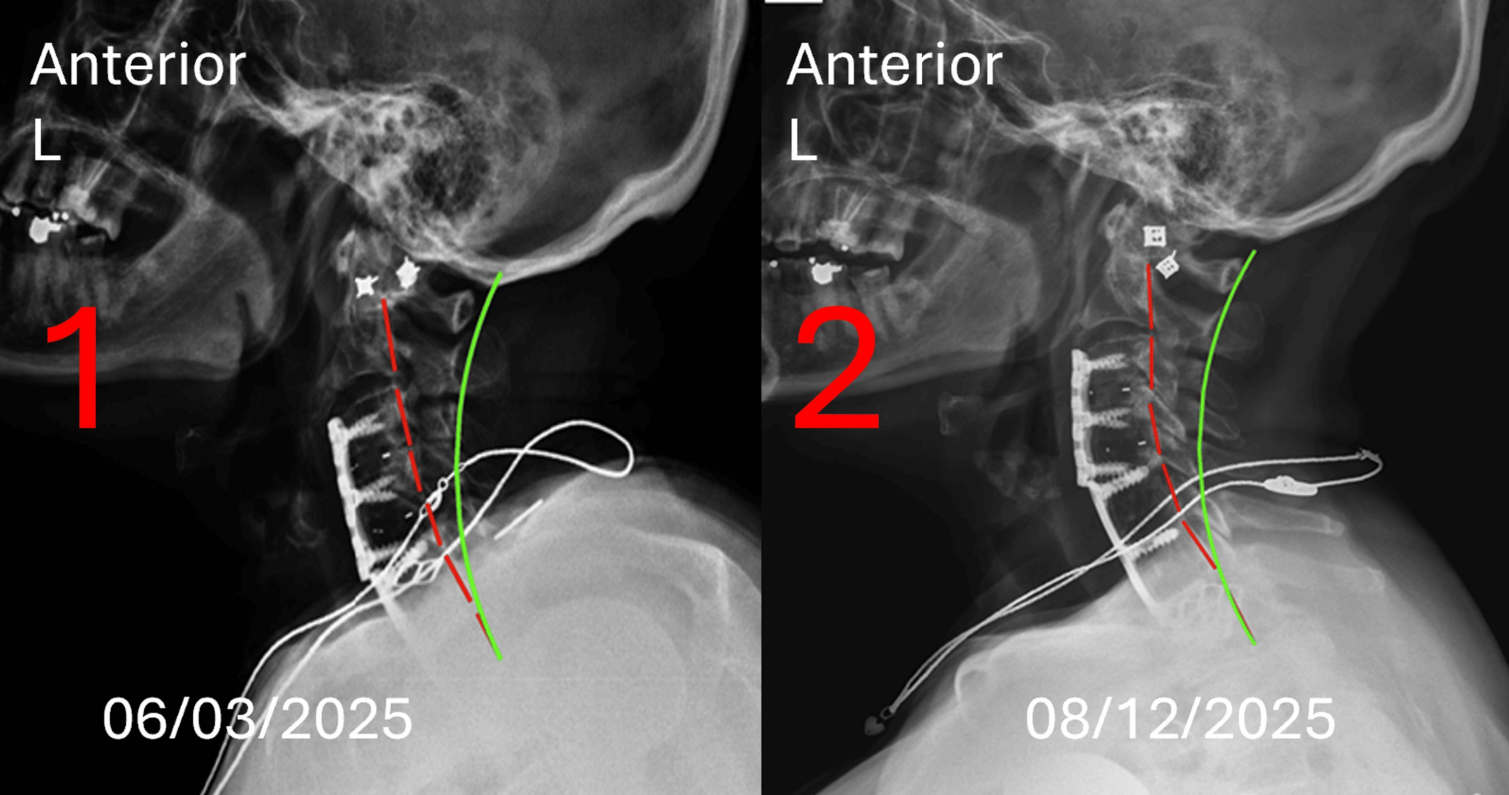
Lateral cervical radiograph compassion The green line depicts the expected alignment of the posterior longitudinal ligament in a normal spine. The red line represents the subjects' alignment as defined by George’s line, reflecting deviation of the posterior longitudinal ligament. The red lines represent the actual posterior tangent lines of the C2-T1 vertebrae using the Harrison posterior tangent method on the lateral cervical radiograph. The green lines represent the ideal spine model. (1) X-ray image represents the initial presentation to the office on 06/03/2025. Pre-treatment neutral lateral cervical radiograph with a cervical curvature ARA C2-C7 measuring -20.4° (ideal measured at -42°, normal is -34°, and pain threshold is -20°) and anterior head translation C2-C7 measuring 21.4 mm (ideal measured at 0 mm, normal is 10 mm, and pain threshold is 20 mm). (2) X-ray image represents the day of discharge from the office on 08/12/2025. Post-treatment neutral lateral cervical radiograph with ARA C2-C7 measuring -23.2° and C2-C7 measuring 19.9 mm. The Absolute Rotation Angle (RRA) tool is a radiographic measurement method used to assess the total angle of spinal curvature.

Lateral Cervical radiographs revealed a persistent anterior head translation, decreased cervical lordosis, and evidence of post-surgical degenerative changes (Figure 3).

The physical examination demonstrated clear postural asymmetry with measurable restrictions in cervical and thoracic range of motion. Focal segmental tenderness that reliably reproduced the presenting symptoms. Neurological examination identified objective abnormalities congruent with the patient’s clinical complaints. This included altered sensorimotor responses and symptom reproduction during targeted positional and functional testing. Radiographic assessment supported these clinical findings by revealing structural and alignment abnormalities corresponding with both the physical and neurological examinations. This indicates a neurologically driven symptom pattern rather than an isolated soft-tissue etiology.

**Figure 4:**
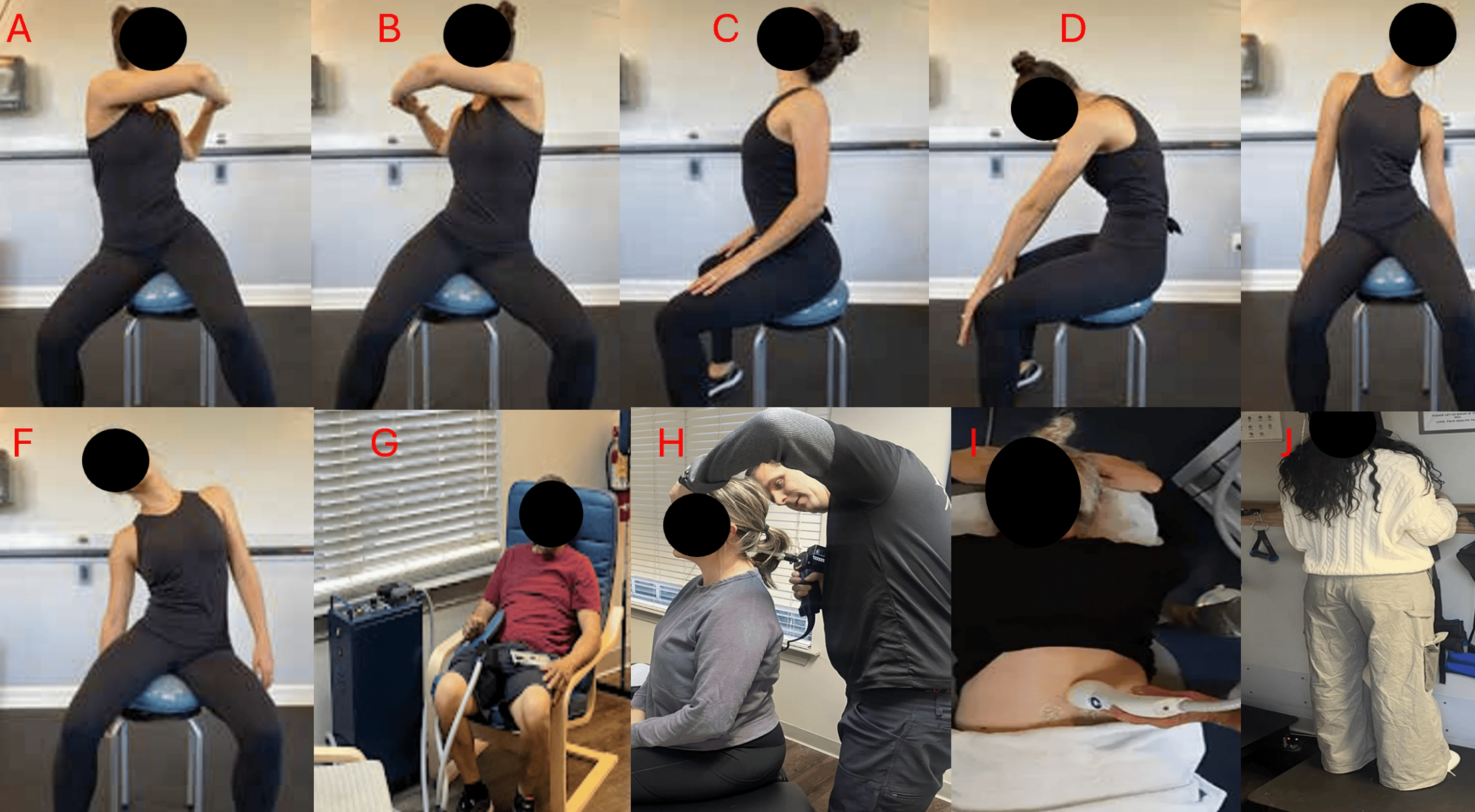
Therapeutic exercises for structural spinal rehabilitation Panels A-F demonstrates active Rehab Chair warm-up exercises (Exercise Therapy), to increase disc metabolism and flexibility through motion. Panel G demonstrates PEMF therapy to modulate pain and promote musculoskeletal healing via enhancement of cellular repair mechanisms and anti-inflammatory effects. Panel H represents adjusting of the cervical spine. Panel I represents ESWT therapy to reduce inflammation, decrease pain, improve blood flow, and accelerate healing of the treatment area, lumbar region. Panel J represents Whole Body Vibration.

The study was conducted at a private integrative rehabilitation clinic located in Charlotte, North Carolina. The facility specializes in non-surgical spine care. It employs a multidisciplinary focus on structural correction, electromedical therapies, and regenerative modalities. The data collection period spanned from June 03, 2025, to August 12, 2025, with a total of 23 visits logged over approximately three months. Initial Motor Vehicle Collision was January 06, 2025. The setting was particularly well-suited for the current investigation, as it provided access to the full suite of therapies required for the conservative structural approach outlined in the study: pulsed electromagnetic field (PEMF) therapy, extracorporeal shockwave therapy (ESWT), and spinal alignment correction using CLEAR Institute protocols [10] (Figure 4).

Spinal structural rehabilitation refers to a conservative, multimodal approach aimed at restoring abnormal spinal alignment and biomechanics. This is done through targeted therapeutic interventions rather than symptom suppression alone. This model emphasizes correction of spinal posture and load distribution through individualized exercises, traction-based interventions, and manual therapies tailored to the patient’s structural findings. The primary objective is to improve neuromuscular function, reduce pathological mechanical stress on spinal tissues, and support durable structural stability over time.

Treatment included PEMF therapy, which was applied using a clinical-grade electromagnetic therapy device calibrated to deliver low-frequency signals shown in prior studies to reduce inflammation and enhance tissue repair [11]. A higher frequency and lower intensity setting was utilized for the acute findings. ESWT was applied to the lumbar and gluteal myofascial tissues to promote local circulation and modulate nociceptive signaling. Five hundred waves per session were used at 2.5 Hz with an average energy level of 7. This is based on protocols adapted from existing literature on mechanical stimulation in chronic spinal pain [12]. Structural spinal correction was conducted using adjusting and neuromuscular retraining techniques derived from the CLEAR Institute protocols, which target spinal alignment restoration through their “mix, fix, and set” protocols of the CLEAR Institute.

At each visit, treatment components were performed sequentially in accordance with the CLEAR Institute's “mix, fix, and set” framework. The total session duration is approximately 60 minutes. The “mix” phase consisted of spinal mobility exercises intended to prepare the tissues for care. This is followed by the “fix” phase, which applies mechanical interventions aimed at improving spinal alignment. The “set” phase focused on rehabilitative procedures designed to reinforce and stabilize the achieved alignment. PEMF was utilized on each visit for 20 minutes. Shockwave was used a total of nine times, weekly, applied to the lumbar region. Adjustments were performed at every visit following CLEAR Institute protocols. Whole body vibration was performed at each visit for 10 minutes. 

Results

The subject did not receive treatment at least 24 hours prior to reexamination to minimize the potential influence of residual effects from a prior treatment session. The subject no longer needed the cane to ambulate and had an unassisted, steady gait. The FRI showed significant improvement with an initial score of 22 and a final score of 11. The subject stated she was no longer taking prescription pain medications. The subject reported complete resolution of the disabling radiating pains into the lower back and left lower extremity. The subject stated they could work without pain. The subject reported they were ready to go dancing, an activity she thought she would not be able to return. The subject reported a marked improvement in cervical range of motion. Dermatomes and myotomes were reported to all be within normal limits (see Table 1).

Radiographic results: Imaging was performed by the same physician who conducted the initial assessment, with radiographic positioning, exposure parameters, and acquisition techniques replicated to ensure consistency. An increase in cervical lordosis was measured on the lateral cervical radiograph at 13.7%, from -20.4 degrees to -23.2 degrees, N=-40.0. A decrease in anterior head translation was measured by 7.0%, from 21.4 mm to 19.9 mm, N=0-20.0mm. A decrease in lumbar lordosis was measured on the lateral lumbar radiograph by 11.4%, from -27.1 degrees to -24.0 degrees, N=-40. A decrease in anterior L1-S1 translation was measured by 32.9%, from 44.7 mm to 30.0 mm, N=0-20 mm. The AP lumbar radiograph measured an improved right translation of 12.0%, -8.3 mm to -7.3 mm, N=0.

## Discussion

The present case report demonstrates the successful resolution of refractory lumbar pain in a 66-year-old female subject with a history of prior lumbar surgery following a motor vehicle collision. The results support an emerging theoretical framework that emphasizes the restoration of spinal alignment as a key component of long-term symptom resolution. The outcomes of this case contribute novel insights while reinforcing some established principles in spine rehabilitation research.

The clinical trajectory observed aligns with prior literature, suggesting that persistent lumbar pain is multifactorial in nature. This appears particularly relevant in cases involving prior lumbar surgery or trauma induced dysfunction. This often involves structural misalignment, chronic inflammation, and neuromuscular imbalance [13]. The subject in the present case exhibited both post-surgical spinal degeneration and trauma-induced exacerbation. This suggests that mono-therapeutic approaches targeting only pain or inflammation would likely be insufficient. Pulsed electromagnetic field therapy provides anti-inflammatory and bio-modulatory effects. Extracorporeal shockwave therapy delivers mechanical and regenerative stimulation, and spinal correction aims to reestablish proper biomechanical integrity.

The observed reduction in pain and disability aligns with prior research demonstrating the efficacy of PEMF in modulating inflammatory cytokine expression and promoting tissue repair. Nelson et al. reported significant pain reduction in chronic low back pain patients treated with PEMF over an 8-week course [14]. Though their population did not include elderly post-surgical cases, the biological rationale remains comparable. The subject in the present study showed similar time-bound improvements. This suggests that PEMF may remain effective even in complex degenerative or post-traumatic spinal conditions.

The mechanical acoustic stimulation provided by ESWT has shown to trigger neoangiogenesis, modulate nociceptor activity, and promote local healing [12]. In comparative trials, patients receiving ESWT reported statistically significant reductions in chronic lumbar pain. These studies often exclude geriatric or surgically altered populations [15]. The present case broadens the scope of ESWT application, demonstrating tolerability and efficacy in an elderly subject.

Persistent post-traumatic spinal pain in older adults is frequently attributed to irreversible degenerative or postsurgical pathology. This often prompts escalation toward invasive management. Altered spinal alignment and postural deviations increase abnormal mechanical loading across multiple pain-sensitive tissues. This may contribute to adult spinal deformity progression and, in some cases, the perceived need for surgical intervention [16]. The subject’s improvement suggests that biomechanical contributors to pain may remain modifiable despite age, prior surgery, and trauma.

Thoracic scoliosis likely played a contributory role in this subject's lumbar symptomatology. Spinal deformity is known to produce global compensatory mechanics rather than regionally isolated dysfunction. Prior studies have demonstrated a high prevalence of abnormal segmental mechanics throughout the spine in patients with scoliosis. This supports a whole-spine biomechanical model of pain generation [17]. Non-surgical, structurally focused interventions have been shown to improve alignment and clinical outcomes in scoliosis populations when care is directed toward restoring spinal balance and neuromuscular control [18,19]. Although much of this literature involves adolescent cohorts. The biomechanical principles remain applicable to adults with established deformity and degenerative change.

A novel component of the treatment protocol was the integration of structural spinal rehabilitation. The theoretical basis here is grounded in the concept of spinal biomechanics as a determinant of chronic pain and neuroinflammation. In this study, radiographic documentation of improved cervical and lumbar alignment paralleled reductions in self-reported pain and disability. This provides compelling support for the biomechanical theory of spinal pain. This suggests that alignment restoration may not only reduce mechanical load but also contribute to neuromuscular re-coordination and inflammatory downregulation. This is particularly relevant in the context of the brain-spine axis. Nociceptive input from biomechanically unstable regions is thought to perpetuate central sensitization [20]. If structural correction interrupts this feedback loop, it may represent a significant theoretical advance in the conservative treatment of chronic spinal pain.

The results align with existing theories regarding inflammation and spinal mechanics. The simultaneous application of three conservative modalities introduces interpretive complexity. As a result, the relative contribution of each individual intervention cannot be isolated with certainty. The attribution of specific clinical improvements to individual interventions becomes difficult. It is this combination that likely underlies the success of treatment in a patient population typically refractory to standard care.

Several limitations must be acknowledged. As a single case report, this study lacks the statistical power and generalizability required for establishing definitive clinical guidelines. The improvements observed may be influenced by subject-specific factors such as high compliance, placebo effect, or spontaneous recovery. The absence of a control or comparative group limits our ability to parse the relative contributions of each intervention. A temporal limitation in follow-up is a limiting factor. 

The findings presented lay the groundwork for a paradigm shift in the conservative management of complex lumbar pain. Future research should aim to conduct controlled trials assessing the efficacy of multimodal conservative interventions versus standard care. This case supports an integrative approach that appears to demonstrate how refractory lumbar pain, particularly in a vulnerable population, can be approached non-invasively.

## Conclusions

This case report demonstrated improvements in subjective and objective measures following conservative, multimodal structural spinal rehabilitation in a subject who failed to improve. This case adds to the literature as a foundational stepping stone toward such research, highlighting a feasible, non-invasive pathway for addressing a highly complex clinical scenario.
